# Duodenal Adenocarcinoma Is Characterized by Acidity, High Infiltration of Macrophage, and Activated Linc01559–GRSF1 Axis

**DOI:** 10.3390/biomedicines13071611

**Published:** 2025-06-30

**Authors:** Xinxin Huang, Ying Shi, Zekun Liu, Yihang Wu, Xiaotong Luo, Dongwen Chen, Zhengyu Wei, Chong Chen, Huaiqiang Ju, Xiaojian Wu, Xuanhui Liu, Zhanhong Chen, Peishan Hu

**Affiliations:** 1 Department of General Surgery (Colorectal Surgery), The Sixth Affiliated Hospital of Sun Yat-sen University, Sun Yat-sen University, Guangzhou 510630, China; huangxx97@mail2.sysu.edu.cn (X.H.); wuyh88@mail2.sysu.edu.cn (Y.W.); luoxt36@mail.sysu.edu.cn (X.L.); chendw25@mail.sysu.edu.cn (D.C.); chench526@mail2.sysu.edu.cn (C.C.); wuxjian@mail.sysu.edu.cn (X.W.); 2 Guangdong Provincial Key Laboratory of Colorectal and Pelvic Floor Diseases, Guangdong Institute of Gastroenterology, The Sixth Affiliated Hospital of Sun Yat-sen University, Sun Yat-sen University, Guangzhou 510630, China; shiying193143@163.com (Y.S.); weizhy28@mail2.sysu.edu.cn (Z.W.); 3 Department of Radiology, Sun Yat-sen University Cancer Center, Sun Yat-sen University, Guangzhou 510220, China; liuzk5@mail.sysu.edu.cn; 4 Sun Yat-sen University Cancer Center, State Key Laboratory of Oncology in South China, Guangdong Provincial Clinical Research Center for Cancer, Sun Yat-sen University, Guangzhou 510220, China; juhuaiq@mail.sysu.edu.cn; 5 Department of Medical Oncology and Guangdong Key Laboratory of Liver Disease, The Third Affiliated Hospital of Sun Yat-sen University, Sun Yat-sen University, Guangzhou 510630, China

**Keywords:** acidic microenvironment, duodenal adenocarcinoma, *Linc01559*, GRSF1

## Abstract

**Background**: Duodenal adenocarcinoma (DA) is often insidious due to the low rate of early diagnosis and because the mechanisms that underlie its malignant progression are poorly understood. The tumor microenvironment (TME) plays a crucial regulatory role in promoting tumor malignancy. Hence, this study aimed to identify novel biomarkers for early diagnosis and potential therapeutic targets for DA. **Methods**: Surgical resection samples and normal tissues from DA patients were collected for RNA sequencing (RNA-seq). The characteristics of TME in DA patients were analyzed, and the differentially expressed long non-coding RNAs (lncRNA) were screened. Functional experiments were performed to verify the relationship between *Linc01559*, G-rich sequence binding factor 1 (GRSF1), and tumor malignant phenotype. **Results**: The present study revealed that DA exhibits a significantly upregulated expression of acidic environment markers and a high degree of macrophage infiltration. Further investigation revealed that macrophages upregulate the expression of the long noncoding RNA, *Linc01559*, in DA through the STAT3/c-MYC signaling pathway, thereby promoting malignant phenotypes such as invasion, metastasis, tumor stemness, and apoptosis. The interaction between GRSF1 and *Linc01559* was subsequently confirmed using RNA pulldown-mass spectrometry. It was further revealed that Linc01559 promotes the malignant phenotype of duodenal cancer cells through its interaction with GRSF1. **Conclusions**: These findings demonstrate that the acidic microenvironment influences the phenotype of DA by regulating the *Linc01559*–GRSF1 axis. Therefore, these findings provide potential targets for the early detection and treatment of DA.

## 1. Introduction

Duodenal adenocarcinoma (DA) is one of the most common malignant tumors of the small intestine, accounting for approximately 50% of all small intestinal malignancies [1,2]. With advancements in imaging techniques, the early diagnostic rate of this disease has improved. The pathogenesis of duodenal cancer is complex and involves multiple factors, including genetic predisposition, environmental influences, and chronic diseases [3]. At present, the treatment for duodenal cancer relies primarily on surgical resection, which is the preferred approach for early-stage cases. For locally advanced or metastatic cases, chemotherapy and radiotherapy are commonly used as adjuvant treatments [4]. Additionally, targeted therapies against Kirsten ratsarcoma viral oncogene homolog (KRAS), Human Epidermal Growth Factor Receptor 2 (HER2), Tumor Protein 53 (TP53), and Adenomatous polyposis coli (APC) are under investigation [5,6]. However, owing to the subtle early clinical symptoms and frequent local invasion and distant metastasis, the 5-year survival rates for patients with stage III and IV are 31.4% and 11.9%, respectively [3]. Identifying specific biomarkers for the diagnosis of duodenal cancer and elucidating the pathogenesis of duodenal cancer will aid in the early detection and treatment of this disease.

Researchers have aimed to identify the genetic alterations and signaling pathways that underlie duodenal adenocarcinoma using high-throughput sequencing and transcriptomics. LncRNAs are central to epigenetic regulation and contribute to gene transcription, post-transcriptional regulation, and post-translational protein modifications. Changes in the expression levels of specific lncRNAs in tumor cells can result in alterations in malignant biological phenotypes, including proliferation, apoptosis, migration, invasion, and angiogenesis [7]. The aberrant expression of lncRNAs, including *MIR17HG*, *SNHG1*, *SP100-AS1*, *DLGAP1-AS2*, *OCC-1*, and *PiHL* [8,9,10,11,12,13], has also been observed in colon cancer. These lncRNAs influence tumor growth regulation, cell cycle progression, invasion, and metastasis by acting as oncogenes or tumor suppressors that contribute to tumor progression and drug resistance. However, the effects and mechanisms by which TAMs influence the lncRNA expression profile in duodenal adenocarcinoma remain unclear. In this study, we found that the intergenic lncRNA *Linc01559* is upregulated in various tumor cells and is closely associated with the initiation and progression of multiple cancer types [14,15,16,17]. In gastric cancer, *Linc01559* promotes cell migration and metastasis by stabilizing E-box-binding zinc finger protein 1 mRNA through the regulation of insulin-like growth factor 2 mRNA-binding protein 2 [17]. Additionally, exosome-derived *Linc01559* from mesenchymal stem cells activates the *PI3K/AKT* pathway, thereby accelerating colorectal cancer development [15]. These findings suggest that *Linc01559* may play an important role in duodenal cancer. We further discovered the protein GRSF1 as an interactor with *Linc01559* through the RNA pull-down experiment.

G-rich sequence binding factor 1 (GRSF1) is an RNA-binding protein involved in transcriptional regulation [18]. Studies have indicated that dysregulated GRSF1 contributes to tumor progression [19]. Notably, GRSF1 enhances the stability of *YY1* mRNA, thereby promoting hepatocarcinogenesis [20]. However, the upstream regulatory mechanisms governing GRSF1 in duodenal cancer remain unclear.

The tumor microenvironment (TME) is a complex ecosystem composed of cells, cytokines, and chemokines that have pivotal roles in tumor initiation and progression. Most solid tumor cells rely on aerobic glycolysis, a phenomenon known as the Warburg effect [21], which supports tumor growth, angiogenesis, and stress response metabolism within the malignant TME [22]. Glycolytic metabolism leads to a significant accumulation of lactic acid, which acidifies the TME. Acidosis is considered a key driver of tumor malignancy and immune evasion [23]. TAMs are critical immune cells within the TME and can be classified into two phenotypic states: The classically activated M1 macrophages and the alternatively activated M2 macrophages. M1 macrophages exhibit pro-inflammatory and anti-tumor properties, whereas M2 macrophages display anti-inflammatory and pro-tumor properties. Owing to their plasticity, TAMs can shift between these dynamically reversible phenotypic states under certain conditions [24]. The recruitment, polarization, and phenotypic conversion of TAMs regulate tumorigenesis and progression by influencing cancer cell proliferation, invasion, metastasis, drug resistance, and immune evasion. For instance, lactate-induced TAM/M2 polarization promotes pituitary adenoma invasion by inducing the expression of C-C motif chemokine ligand 17 [25]. Additionally, lactate secreted by breast cancer cells triggers M2 polarization through the *ERK/STAT3* signaling pathway [26]. However, the impacts of lactate and TAMs in the TME on duodenal cancer progression remain unclear.

In the present study, an RNA sequencing analysis of 11 duodenal adenocarcinoma tissues and their corresponding adjacent normal tissues was conducted. Transcriptome, pathway enrichment, cluster, and immune cell infiltration analyses were also performed. The findings revealed that acidic environment markers were significantly upregulated in patients with duodenal cancer, that macrophages exhibited high infiltration, and that the expression of acidic environment markers was positively correlated with macrophage infiltration. *Linc01559* was also identified as the most significantly upregulated lncRNA and its co-expressed protein-coding genes were explored. Further experiments were conducted to determine the mechanism by which the *Linc01559*–GRSF1 axis promotes the malignant phenotype of duodenal cancer. The present study aimed to identify novel molecular targets, to potentially improve the treatment strategies available for patients with duodenal cancer.

## 2. Materials and Methods

### 2.1. Cells and Cell Culture

Human duodenal adenocarcinoma cell lines (WDC-1, HuTu-80) were obtained from the American Type Culture Collection (Manassas, VA, USA) and cultured in RPMI 1640 (Gibco, New York, NY, USA) supplemented with 10% FBS (Bioexploper, Boulder, CO, USA) and penicillin/streptomycin (Gibco). All cells tested negative for mycoplasma contamination and were identified by STR fingerprinting before use. The reagents used in this study are shown in Appendix A, Table A4.

### 2.2. RNA-Seq

Total RNA was extracted from tumor tissues or cells using Trizols reagent (Thermo Fisher, Waltham, MA, USA), and then 2 µg of total RNA was taken for strand RNA sequencing library preparation by the SeqHealth Company, London, UK. A principal component analysis (PCA) was performed using R-package FactoMineR (v2.11) based on complete gene expression profiles. A differential expression analysis used R-package DESeq2 (v1.40.2) to extract differentially expressed genes based on the original count expression profile (*p*adj < 0.05, Foldchange ≥ 2). The volcano plot of differentially expressed genes was drawn based on R-package ggplot2(v3.5.1) (*p*adj < 0.05, Foldchange ≥ 2). Differentially expressed lncRNAs and their related mRNAs’ expression heatmaps were plotted using an R-package pheatmap(v1.0.12). The differentially expressed genes between the high–low expression groups of GRSF1 were analyzed using R-package Limma (v3.56.2) (*p*adj < 0.05, Foldchange ≥ 2), and some significant genes were displayed using the violin plot of R-package ggplot2 (v3.5.1).

### 2.3. Enrichment Analysis of Functional Pathways

The correlation between lncRNA and mRNA expression was calculated using basic R function cor.test (*p* < 0.05, R ≥ 0.5), and a cancer hallmarks pathway enrichment analysis was performed for the significantly correlated mRNAs using a cluster Profiler [27]. In addition, based on the correlation results, the enrichment of 50 cancer hallmarks from MSigDB [28] was analyzed based on a GSEA algorithm (*p* < 0.05), and some of the significantly enriched results were plotted using an R-package enrichplot (v1.22.0). In the analysis part of GRSF1, the mRNAs were analyzed by GSEA (*p* < 0.05) based on the correlation with the expression of GRSF1, and the significantly enriched cancer hallmarks were visualized using enrichplot.

### 2.4. Immune Infiltration Analysis

In terms of the immune infiltration analysis, the tool Cibersort [29] was used to decompose the complete gene expression profile to obtain the abundance of immune cells, R-package ggplot2 was used to visually display the differences in the abundance of global immune cells as well as the expression levels of acidic characteristic genes between tumor and adjacent tissues, and the differences in the expression levels of acidic characteristic genes between high and low lncRNA expression groups. The statistical significance of the difference between groups was tested using a Wilcoxon test (*p* < 0.05). The correlation heatmaps among differential lncRNAs, macrophage, and acid characteristic genes were plotted using R-package corrplot (v0.94) (*p* < 0.05), and the scatterplots of expression correlation among them were drawn using R-package ggplot2.

### 2.5. RNA Pulldown

The Linc01559 expression plasmid containing a T7 promoter was linearized by PCR amplification, with product verification via agarose gel electrophoresis followed by DNA purification. DNA was collected in vitro and transcribed into RNA. Following the manufacturer’s protocol for the Pierce™ Magnetic RNA-Protein Pull-Down Kit (20164, Thermo Scientific), biotinylated RNA was conjugated to streptavidin magnetic beads through 20 min incubation at room temperature. Tumor cell lysates were subsequently incubated with the RNA–bead complexes at 4 °C for 1 h with constant rotation to facilitate RNA–protein interactions. After six stringent wash cycles, bead-bound proteins were eluted in 5 × SDS loading buffer through 10 min denaturation at 95 °C. Mass spectrometric analysis of the eluates identified Linc01559-interacting proteins, with a subsequent bioinformatic analysis prioritizing the most abundantly bound candidates.

### 2.6. RNA-Binding Protein Immunoprecipitation (RIP)

RIP assays were conducted using the Magna RIP™ RNA-Binding Protein Immunoprecipitation Kit (17-700, Merck Millipore, Burlington, MA, USA). A total of 4 × 10^7^ tumor cells were collected in a centrifuge tube and washed once. Subsequently, 200 µL of prepared RIP complete lysis buffer was added, and the mixture was incubated on ice for 5 min. After vortexing, the lysate was placed at –80 °C for 5 min and then evenly divided into two parts. Protein A/G magnetic beads were prepared concurrently, and 5 µg of either IgG or GRSF1 antibody (54507, Signalway, Greenbelt, MD, USA) was incubated with the beads for 30 min at room temperature with rotation. The lysate was then rapidly thawed, centrifuged at 14,000 rpm for 10 min at 4 °C, and the resulting supernatant was added to the RIP immunoprecipitation buffer containing the bead–antibody complexes. This mixture was incubated overnight at 4 °C with rotation. The eluted RNA was purified, followed by reverse transcription, PCR amplification, and agarose gel electrophoresis to assess RNA-binding efficiency.

### 2.7. RT-qPCR

Total RNA was extracted with an EZ-press RNA Purification Kit (B0004D, EZBioscience, Roseville, MN, USA) and the cDNA was synthesized using a Color Reverse Transcription Kit (A0010CGQ, EZBioscience). Use 2× Color SYBR Green qPCR Master Mix (A0012-R2, EZBioscience) and QuantStudio 6 system (Thermo) for quantitative real-time PCR (qPCR). According to the following reaction program, the first stage was hot start: 95 °C, maintained for 5 min (1 cycle); the second stage was amplification cycle denaturation: 95 °C for 10 s; annealing and extension at 60 °C for 30 s (all 40 cycles); the third stage was melting curve: 60 °C, 60 s; 95 °C for 15 s. mRNA expression was measured by primer-based qPCR (Appendix A Table A1).

### 2.8. Cell Proliferation

Briefly, transfected duodenal cancer cell lines were seeded at a density of 2000 cells per well in 96-well plates. Cell viability was assessed using the Cell Counting Kit-8 (CCK-8, Beyotime, Shanghai, China), and growth curves were generated accordingly. Data shown are presented from at least three independent experiments.

### 2.9. Colony Formation

Following siRNA transfection, tumor cells were trypsinized to generate single-cell suspensions (density: 2000 cells/well in 6-well plates) and maintained in an RPMI-1640 medium (Gibco, New York, NY, USA) supplemented with 10% FBS (Bioexploper). The medium was refreshed every 48 h, with a second siRNA transfection performed on day 3 post-seeding. After 7 days of culture, colonies were fixed with 4% paraformaldehyde (PFA; 1 mL/well, 1 h), washed twice with PBS, and stained with 0.5% crystal violet (1 mL/well, 15 min). Air-dried plates were imaged under standardized conditions, and colony quantification was performed using the ImageJ (v1.53) program.

### 2.10. Cell Migration and Invasion Assays

Cell migration and invasion tests were performed after siRNA treatment with 24-well Transwell Inserts (Corning, New York, NY, USA). After transfection, the cells were cultured in a 5% CO_2_ incubator at 37 °C for 4–6 h. Single cells suspended in a serum-free medium were added to the chamber and a medium containing 10% FBS was added to the bottom. After incubation for 16–20 h, the migrated cells were moved to the surface of the lower membrane, stained with crystal violet, observed under a 10× objective lens, and the images of at least three random fields were taken. The quantization method is as follows: Three equally sized views are randomly selected from the original plots of each group for an artificial cell count. The average cell count for each siScramble test was then normalized to 1 and the cell count for the corresponding group was adjusted accordingly. Then the mean and standard deviation are calculated and shown in a bar chart. A statistical analysis was performed using GraphPad Prism (v8.0) and Student’s *t*-test. The data presented are from at least three separate experiments.

### 2.11. Apoptosis

Cell apoptosis was assessed using the FITC Annexin V Apoptosis Detection Kit (BD Pharmingen, Franklin Lakes, NJ, USA). Following siRNA treatment of WDC-1 and HuTu-80 cells, cells were harvested and washed twice with PBS according to the experimental protocol. The cells were then resuspended in 500 µL of 1× Annexin V binding buffer, followed by the addition of 5 µL Annexin V-FITC and 5 µL propidium iodide (PI) solution. Samples were incubated in the dark at room temperature for 15 min. After incubation, fluorescence signals were detected using a BD LSRFortessa flow cytometer (BD Biosciences, San Jose, CA, USA). Annexin V-FITC and PI staining were used to identify early and late apoptotic cells, respectively. Data were analyzed using FlowJo v10 software (FlowJo LLC, Ashland, OR, USA).

### 2.12. Western Blotting

Cell lysis was performed using a RIPA buffer supplemented with protease and phosphatase inhibitors (Roche, Basel, Switzerland). The RIPA buffer contained 150 mM NaCl, 50 mM Tris-HCl, 0.5% sodium deoxycholate, 200 mM NaF, 1.0% NP-40, and 1 mM EDTA. The lysates were centrifuged at 14,000 rpm for 15 min at 4 °C. Protein concentrations were measured using the BCA Protein Assay Kit (Beyotime, Shanghai, China). The protein loading buffer (Epizyme, Shanghai, China) was added to the lysates, followed by denaturation at 95 °C for 10 min. Equal amounts of protein and pre-stained protein markers (GenStar, Beijing, China) were loaded onto SDS-PAGE gels and separated using an electrophoresis buffer (0.25 M Tris, 1.92 M glycine, 1% SDS). The proteins were transferred to PVDF membranes, which were then blocked with 5% skim milk for 1 h at room temperature. Membranes were incubated overnight at 4 °C with primary antibodies (see Table A3 in Appendix A), followed by incubation with HRP-conjugated secondary antibodies (1:5000 dilution) for 1 h at room temperature. Immunoreactive bands were visualized using the ECL Ultra kit (NCM).

### 2.13. Spheroid Formation Assay

The sphere-forming assay is a commonly used in vitro method to investigate the self-renewal properties of cancer stem cells. A total of 5000 cells were seeded in 96-well ultra-low attachment plates using a DMEM/F12 medium (Gibco) supplemented with 20 ng/mL human recombinant EGF, 10 ng/mL human recombinant FGF, B27 1:50 (Invitrogen, Carlsbad, CA, USA), and 10 µg/mL heparin. The cells were incubated at 37 °C in a humidified CO_2_ incubator with a volume of 100 µL per well. After one week, the total number of tumor spheres larger than 50 µm in diameter in each well was quantified under an inverted microscope (Olympus, Tokyo, Japan).

### 2.14. Construction of GRSF1 Overexpression Cell

The pCDNA3.1(+) vector was used to construct a GRSF1 overexpression plasmid. Full-length coding sequences of GRSF1 (NM_001098477.2 and NM_002092.4) were amplified from a human cDNA library via PCR and subsequently cloned into the multiple cloning site of the pCDNA3.1(+) vector to generate the recombinant plasmid pcDNA3.1-*GRSF1*. The insertion was verified by Sanger sequencing (RIBOBIO). The pcDNA3.1-*GRSF1* plasmid was transfected into HEK293T cells using Lipofectamine 3000 (Invitrogen) to produce lentiviral particles, with the empty vector (pcDNA3.1-empty) serving as a negative control. After 72 h of incubation, viral supernatants were harvested and filtered. WDC-1 cells were transduced with the viral supernatant in the presence of 10 µg/mL polybrene overnight. Stable cell lines overexpressing GRSF1 were selected using puromycin, and overexpression was confirmed by Western blot analysis.

## 3. Results

### 3.1. Macrophage Infiltration and Significant lncRNA Alterations in Duodenal Adenocarcinoma Under an Acidic Environment

**Figure 1:**
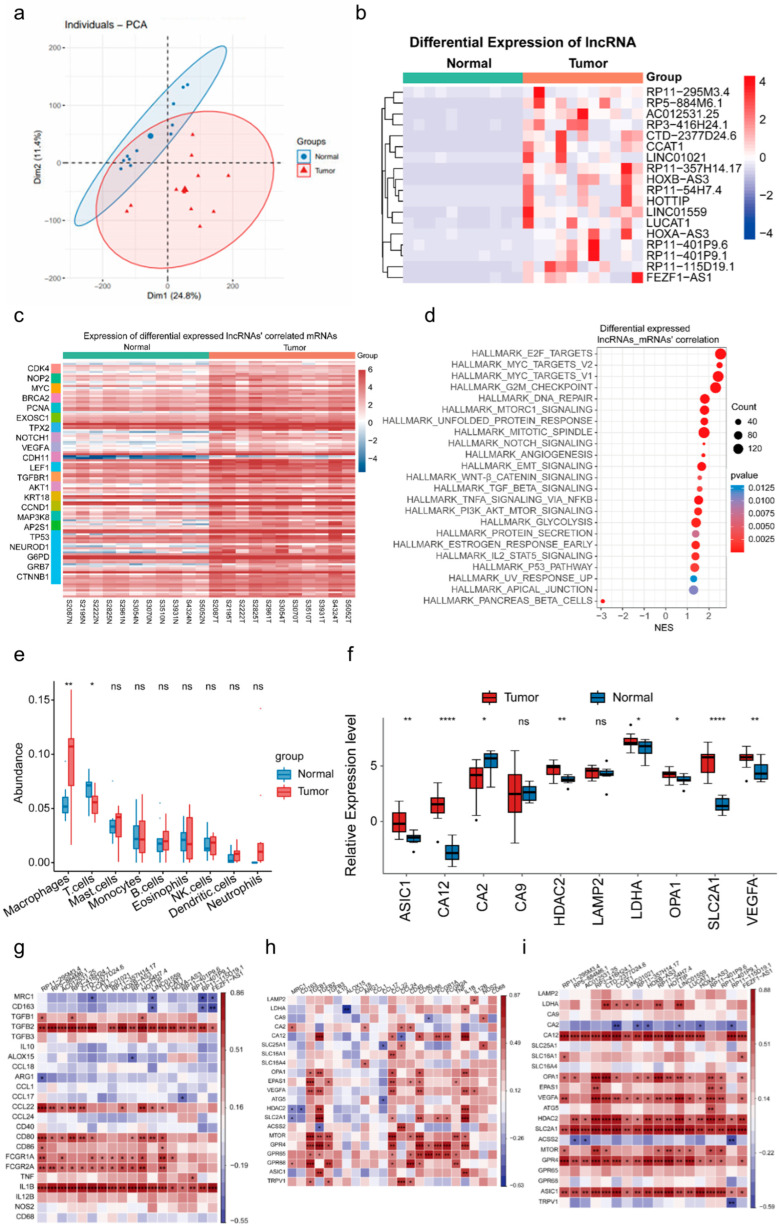
The microenvironment of duodenal adenocarcinoma is characterized by acidity and a high level of macrophage infiltration. (**a**) Principal component analysis (PCA) of transcriptome sequencing data from 11 paired samples of duodenal adenocarcinoma tumor tissues and adjacent normal tissues; (**b**,**c**) heatmap of 18 differentially expressed lncRNAs (**b**) and their correlated mRNAs (**c**) between normal and tumor tissues, as described in Figure 1a Signaling pathways related to these genes are shown in panel C; (**d**) cancer hallmark signaling pathways associated with the genes co-expressed with the differentially expressed lncRNAs; (**e**) infiltration levels of immune cells in normal and duodenal adenocarcinoma tumor tissues; (**f**) mRNA expression levels of acidic environmental markers in normal and duodenal adenocarcinoma tumor tissues; (**g**–**i**) correlation analysis of expression between 18 lncRNAs and macrophage markers (**g**); macrophage markers and acidic environmental markers (**h**); and 18 lncRNAs and acidic environmental markers (**i**); The Pearson correlation coefficient (r) and *p*-values are shown * *p* < 0.05, ** *p* < 0.01, *** *p* < 0.001 and **** *p* < 0.0001.

**Figure A1:**
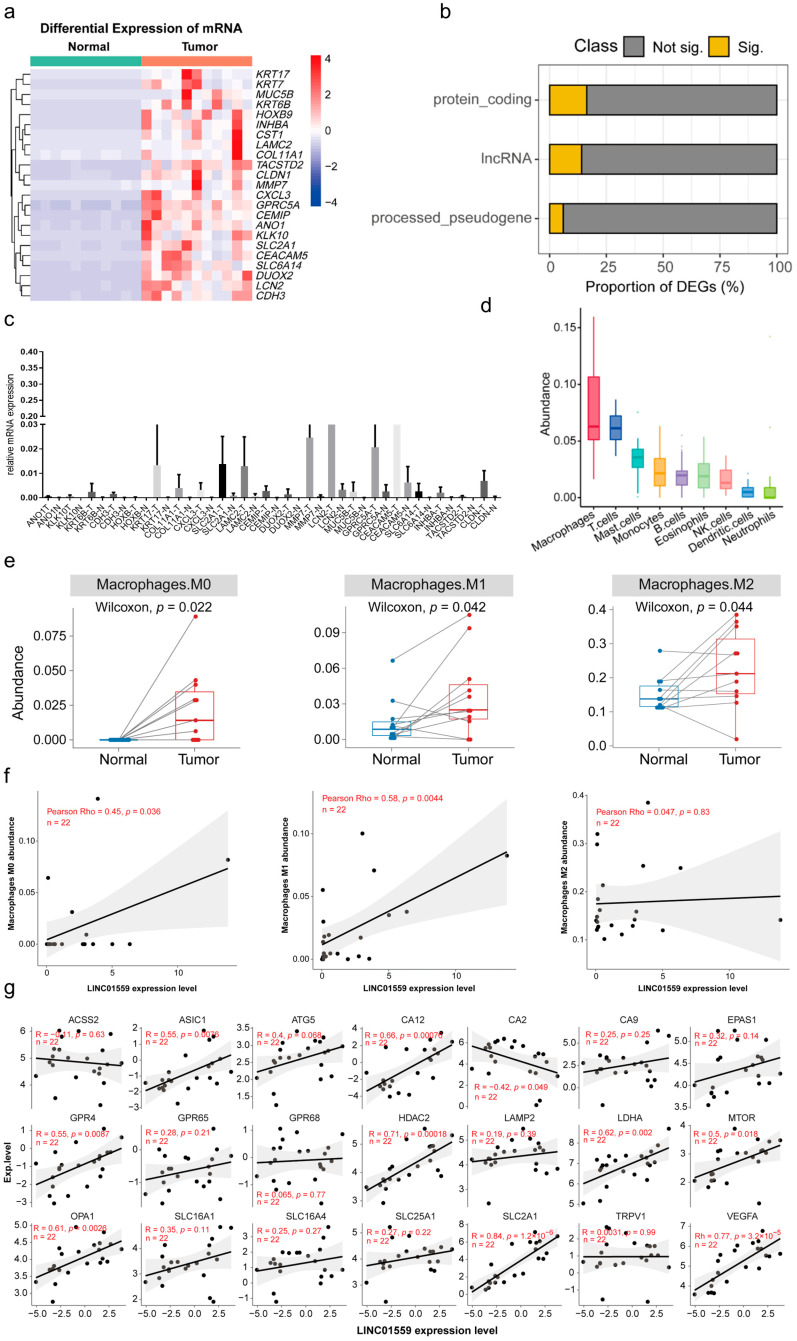
The microenvironment of duodenal adenocarcinoma is characterized by acidity and a high level of macrophage infiltration and Linc01559 expression. (**a**) Heatmap of differentially expressed coding genes between normal and duodenal adenocarcinoma tumor tissues; (**b**) the proportion of protein-coding genes, lncRNAs, and processed pseudogenes in duodenal adenocarcinoma tumor tissues; (**c**) qPCR of RNA expression of differentially expressed coding genes as described in Figure A1 (**a**); (**d**) infiltration levels of immune cells in duodenal adenocarcinoma tumor tissues; (**e**) infiltration levels of macrophages in normal and duodenal adenocarcinoma tumor tissues; (**f**) correlation analysis of the expression between *Linc01559* and macrophages; and (**g**) correlation analysis of the expression between *Linc01559* and acidic environmental markers. Three independent experiments were performed to obtain the data in (**c**). The data are shown as the mean ± SD; the Pearson correlation coefficient (r) and *p*-values are shown.

To investigate the transcriptomic characteristics of duodenal adenocarcinoma and identify markers associated with malignant phenotypes, transcriptome sequencing was performed on 11 pairs of duodenal adenocarcinoma tumor tissues and adjacent normal tissues (Appendix A, Table A2). A quality control analysis via a principal component analysis (PCA) confirmed the distinct heterogeneity between normal and tumor tissues (Figure 1a). A differential gene expression analysis revealed significantly upregulated genes in the tumor tissues compared with normal tissues (Appendix B, Figure A1a), which was further validated using qPCR. Notably, the classification of the differentially expressed genes showed that a marked proportion were lncRNAs (Appendix B, Figure A1b). Numerous studies have demonstrated the critical role of lncRNAs in tumor development and progression. The differential expression analysis confirmed a significant upregulation of lncRNAs in duodenal adenocarcinoma (Figure 1b). A co-expression analysis of these lncRNAs with mRNAs, followed by a pathway enrichment analysis, indicated that the associated signaling pathways were primarily linked to tumor stemness, invasion, metastasis, and immune invasion (Figure 1c,d).

A CIBERSORT analysis was conducted on the tumor sample and adjacent tissue samples to further explore immune cell infiltration. Infiltrating macrophages were the most prominent immune cell type (Figure 1e), with significantly higher infiltration observed in the tumor tissues (Appendix B, Figure A1d–f). TAMs within the tumor immune microenvironment contribute to cancer cell migration and invasion by interacting with cancer or stromal cells through cytokines, chemokines, or growth factors [30]. High macrophage infiltration was identified in the acidic microenvironment of duodenal adenocarcinoma, which is consistent with previous studies on colorectal cancer. Further analysis revealed that, compared with the adjacent normal tissues, the tumor samples exhibited upregulated expression of acidic environment markers, including lactate dehydrogenase A (LDHA), VEGFA, carbonic anhydrase (CA)9, and lysosomal-associated membrane protein 2 (Figure 1f). A correlation matrix analysis revealed positive associations between the highly expressed lncRNAs, macrophages, and acidic environment markers (Figure 1g,h). Additionally, the acidic microenvironment in duodenal adenocarcinoma was significantly correlated with TAMs (Figure 1i). These findings suggest that lncRNAs may serve pivotal roles in the tumorigenesis and progression of duodenal adenocarcinoma. High macrophage infiltration within the acidic TME represents a defining characteristic of the duodenal adenocarcinoma TME.

### 3.2. Linc01559 Is Upregulated in Duodenal Adenocarcinoma and Is Correlated with Acidic Environment Markers

**Figure 2:**
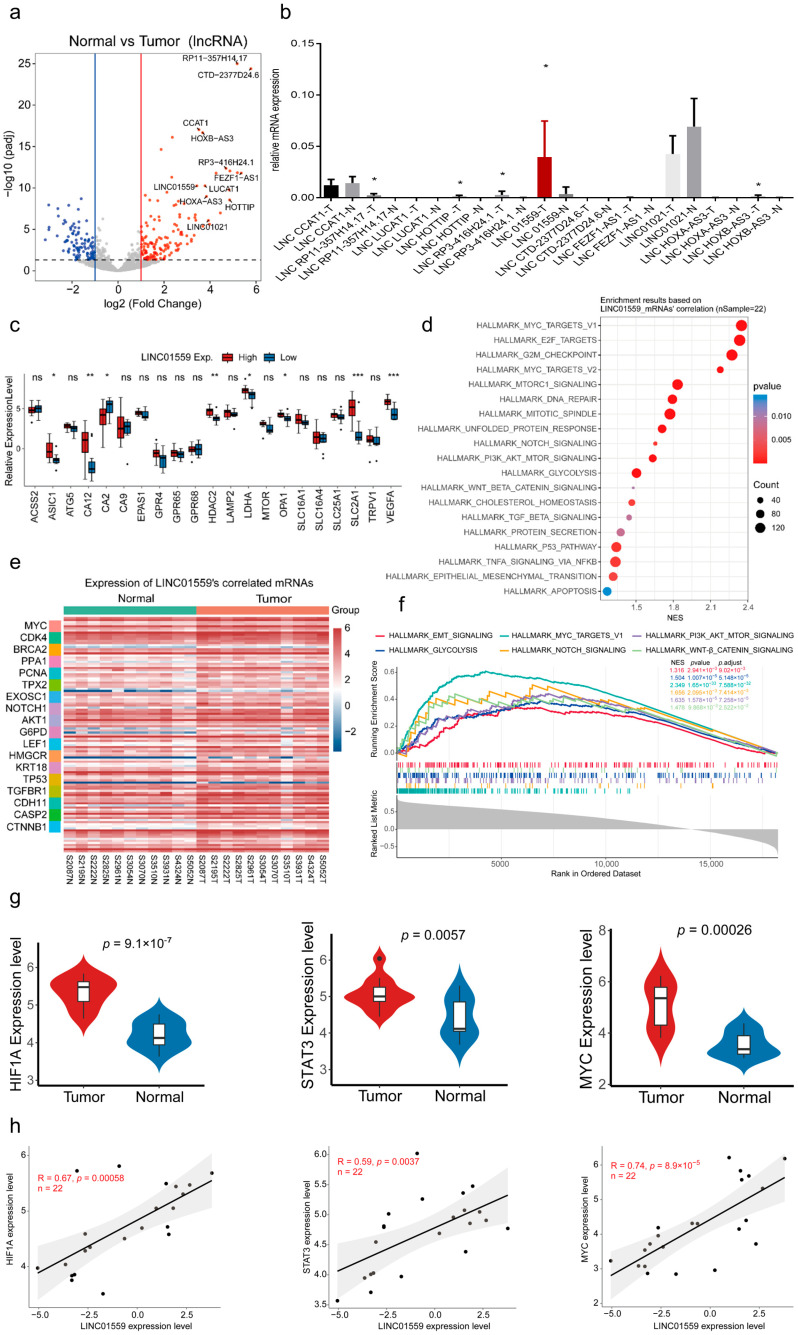
High expression of Linc01559 is positively correlated with an acidic environment and the STAT3-cMYC pathway in duodenal adenocarcinoma. (**a**) The volcano plot shows significantly overexpressed lncRNAs in duodenal adenocarcinoma tumor tissues; (**b**) qPCR of RNA expression ofdifferentially expressed lncRNAs described in Figure 2a; (**c**) mRNA expression levels of acidic environmental markers in *Linc01559* high and *Linc01559* low expression group; (**d**–**f**) heatmap of genes co-expressed with *Linc01559* (**e**) and the cancer hallmark signaling pathways associated with these genes (**d**,**f**) between normal and tumor tissues are shown; (**g**) mRNA expression of HIF1α, STAT3 and c-MYC in normal and tumor tissues; (**h**) correlation analysis of the expression between *Linc01559* and HIF1α, STAT3, c-MYC. Three independent experiments were performed to obtain the data in (**b**); The data are shown as the mean ± SD; the Pearson correlation coefficient (r) and *p*-values are shown * *p* < 0.05, ** *p* < 0.01, and *** *p* < 0.001.

To identify key lncRNAs associated with the malignant phenotypes of duodenal adenocarcinoma, differentially expressed lncRNAs in tumor tissues, compared with normal tissues, were examined using volcano plots (Figure 2a). Subsequent validation via qPCR confirmed that Linc01559 was significantly upregulated in tumor tissues (Figure 2b). Further analysis revealed that patients with high *Linc01559* expression exhibited significantly upregulated expression of acidic environment markers, including acid-sensing ion channel subunit 1, CA12, histone deacetylase 2, LDHA, OPA1, solute carrier family 2 member 1, and VEGFA (Figure 2c). A Pearson correlation coefficient analysis revealed a positive correlation between *Linc01559* and these acidic environment markers (Appendix B, Figure A1g). Notably, *Linc01559* was also significantly associated with macrophage markers (Appendix B, Figure A1f).

**Figure A2:**
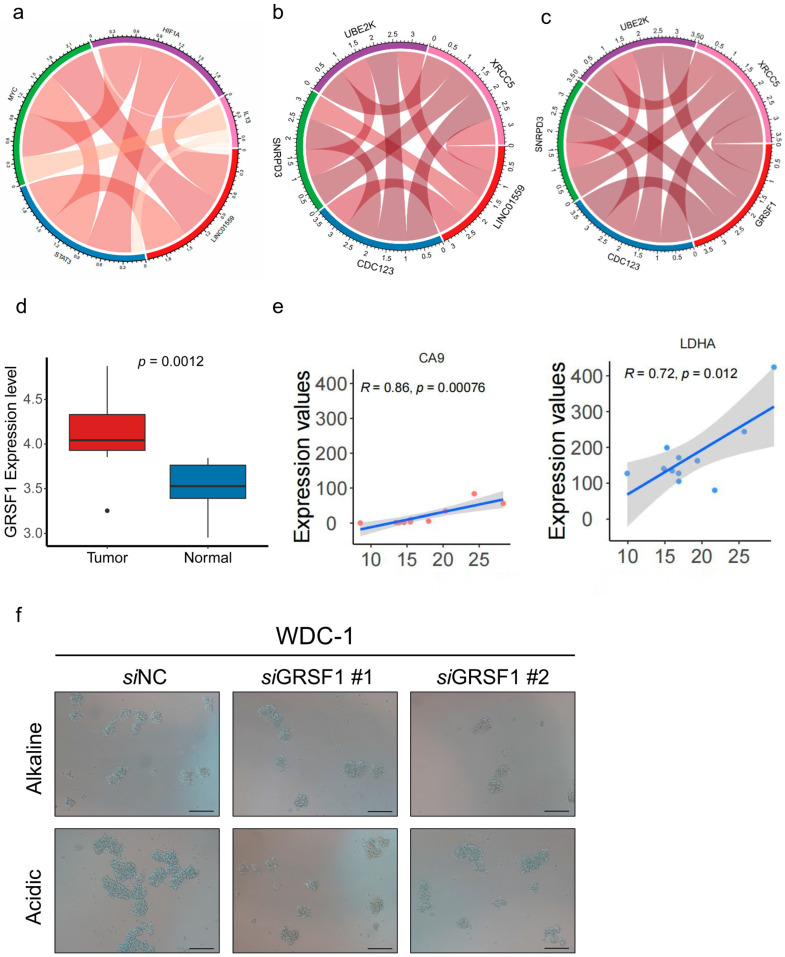
(**a**) Correlation analysis among *Linc01559*, IL13, HIF1A, c-MYC, and STAT3 in normal and tumor tissues; (**b**,**c**) correlation analysis among *Linc01559* (**b**), GRSF1 (**c**), and stemness markers; (**d**) mRNA expression levels of GRSF1 in normal and duodenal adenocarcinoma tumor tissues; (**e**) correlation analysis of the expression between GRSF1 and acidic environmental markers CA9, LDHA in tumor tissues.; and (**f**) representative images of tumor spheres (larger than 50 µm in diameter) formed after knockdown of GRSF1 in WDC-1 duodenal cancer cells under acidic and alkaline conditions. Scale bar: 100 µm. The Pearson correlation coefficient (r) and *p*-values are shown.

The protein-coding genes co-expressed with Linc01559 and their associated signaling pathways were next investigated. A cancer hallmark enrichment analysis revealed pathways such as *MYC*, G2M checkpoint, DNA repair, Notch, *AKT*, glycolysis, Wnt, *TGF-β*, EMT, and apoptosis, which are closely linked to tumor stemness, invasion, metastasis, apoptosis, and metabolism (Figure 2e). These findings were further validated through a Gene Set Enrichment Analysis (GSEA) (Figure 2f). Glycolysis was prominently featured in the pathways associated with *Linc01559*, as was the hypoxia-inducible factor 1 subunit α(HIF1α), a key regulator of glycolysis. Increased *HIF1α* expression induces glycolysis, immune escape, metastasis, and angiogenesis in hypoxic tumors [31]. Our previous studies demonstrated that acidic environments promote the malignant phenotypes of gastric and colorectal cancer by regulating macrophage-secreted cytokines and influencing the STAT3/c-MYC axis in tumor cells. The HIF1α, STAT3, and MYC expression levels in duodenal adenocarcinoma were analyzed alongside their correlation with *Linc01559*. It was found that HIF1α, STAT3, and MYC were highly expressed in tumor tissues (Figure 2g) and were positively correlated with *Linc01559* expression (Figure 2h and Appendix B, Figure A2a). These results suggest that the acidic TME promotes the upregulation of *Linc01559* in duodenal adenocarcinoma by influencing macrophage activation, which in turn drives malignant phenotypes such as stemness, invasion, metastasis, and apoptosis.

### 3.3. Linc01559 Knockdown Inhibits the Invasion, Migration, and Tumor Stem Cell Phenotype of Duodenal Adenocarcinoma

**Figure 3:**
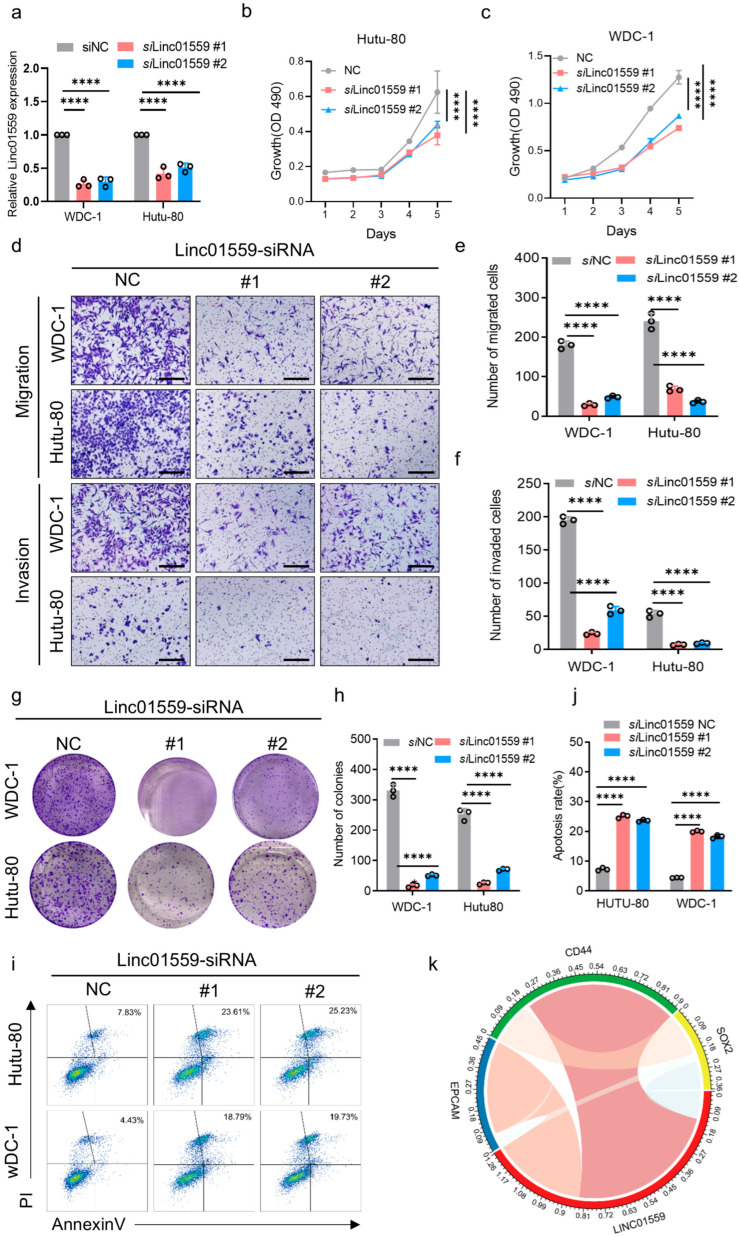
*Linc01559* promotes the malignant phenotype of duodenal adenocarcinoma cells. (**a**) qPCR was used to verify the expression of Linc01559 in duodenal adenocarcinoma cells treated with control and *Linc01559*-targeting siRNA; (**b**,**c**) cell viability of control and *Linc01559*-knockdown duodenal adenocarcinoma cells is shown, with the IC50 indicated by a dotted line; (**d**–**f**) representative images (**d**) and quantified data (**e**,**f**) from the transwell assay demonstrate the migration and invasion ability of control and *Linc01559*-knockdown duodenal adenocarcinoma cells. (scale bar,100 µm); (**g**,**h**) representative images (**g**) and quantified data (**h**) of the colony formation assay illustrate the stemness characteristics of control and *Linc01559*-knockdown duodenal adenocarcinoma cells; (**i**,**j**) representative images (**i**) and quantified data (**j**) from flow cytometry analysis show the apoptosis of control and *Linc01559*-knockdown duodenal adenocarcinoma cells; (**k**) correlation analysis between *Linc01559* and stemness marker EPCAM, CD44, and SOX2. Three independent experiments were performed to obtain the data in (**a**,**e**,**f**,**h**,**j**). The data are shown as the mean ± SD; the Pearson correlation coefficient (r) and *p*-values are shown **** *p* < 0.0001.

To further elucidate the role of *Linc01559* in the malignant phenotype of duodenal adenocarcinoma cells, *Linc01559* expression was knocked down in the Hutu-80 and WDC-1 cell lines using siRNA (Figure 3a). Cell proliferation was significantly reduced following *Linc01559* knockdown, as assessed via the CCK-8 assay (Figure 3b,c). Additionally, invasion and migration assays demonstrated that *Linc01559* knockdown markedly inhibited the invasive and migratory capacities of duodenal adenocarcinoma cells (Figure 3d–f). Plate colony experiments further revealed a significant reduction in the colony-forming ability (Figure 3g,h). Furthermore, an apoptosis analysis indicated that knocking down *Linc01559* expression promoted the apoptosis of duodenal adenocarcinoma cells (Figure 3i,j). An analysis of the tumor stem cell markers revealed a significant positive association between Linc01559 expression and the expression of the markers epithelial cell adhesion molecule (EPCAM), CD44, ubiquitin-conjugating enzyme E2 K (UBE2K), X-ray repair cross-complementing 5(XRCC5), small nuclear ribonucleoprotein D3 polypeptide (SNRPD3), and cell division cycle 123 (CDC123) (Figure 3k and Appendix B, Figure A2b). These findings suggest that *Linc01559* knockdown inhibits the malignant phenotypes, including invasion, migration, and tumor stemness, while promoting apoptosis in duodenal adenocarcinoma cells.

### 3.4. GRSF1 Is a Target Protein That Interacts with Linc01559

**Figure 4:**
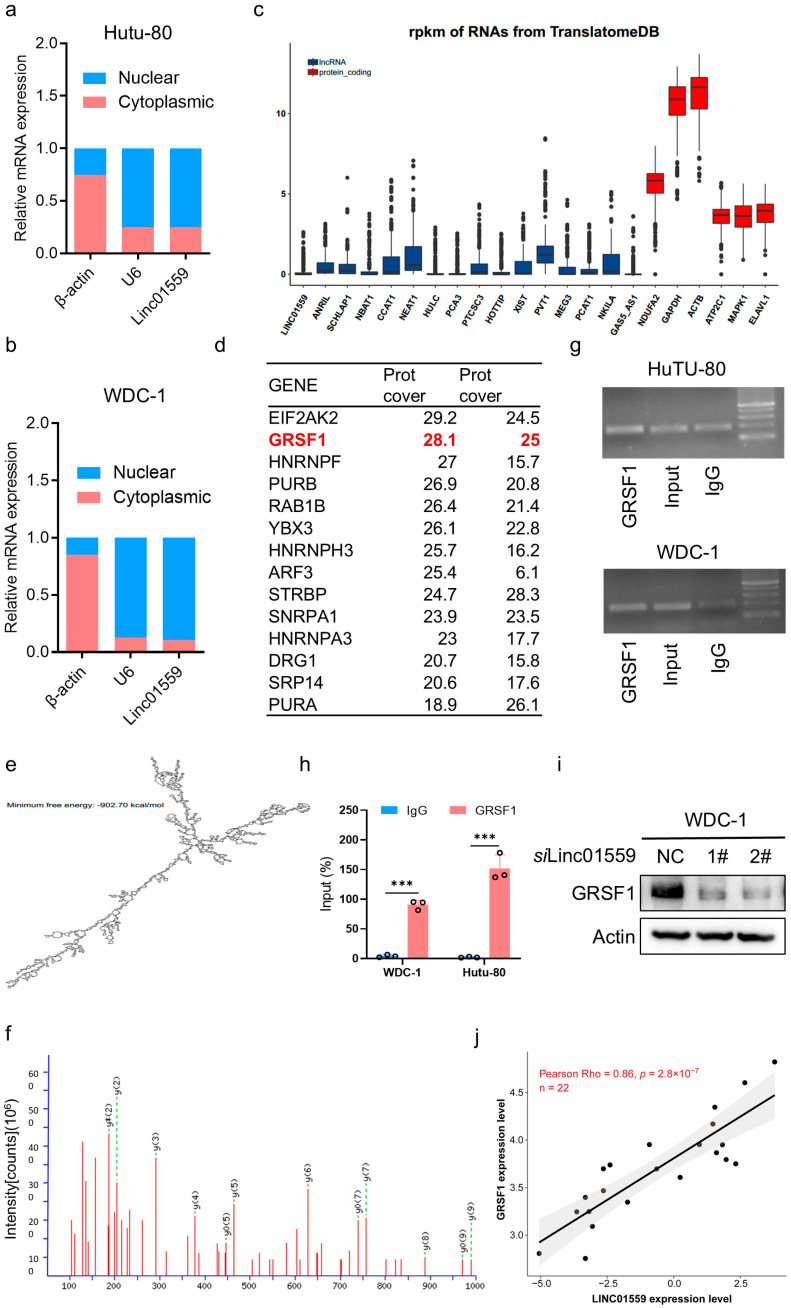
GRSF1 is the target protein interacting with *Linc01559*. (**a**,**b**) qPCR analysis of the RNA expression levels of β-actin, U6, and Linc01559 in the nuclear and cytoplasmic fractions of HuTu-80 (**a**) and WDC-1 (**b**) cells; (**c**) translatome DB analysis of the protein expression of lncRNAs as described in Figure 2a and mRNAs; (**d**) RNA pull down of the detected protein that interacted with Linc01559; (**e**). The secondary structure diagram of *Linc01559*; (**f**) mass spectrometry results as described in Figure 4d; (**g**,**h**) the interaction between GRSF1 and *Linc01559* is detected by RIP (**g**), the immunoprecipitated RNA is quantified by qPCR(h); (**i**) western blot of the protein expression of GRSF1 in control and *Linc01559*-knockdown duodenal adenocarcinoma cells; (**j**) correlation analysis of the expression between *Linc01559* and GRSF1. Three independent experiments were performed to obtain the data in (**a**,**b**,**h**). The data are shown as the mean ± SD; the Pearson correlation coefficient (r) and *p*-values are shown *** *p* < 0.001.

The regulatory effect of an acidic environment on the malignant phenotype of duodenal adenocarcinoma cells through *Linc01559* was preliminarily confirmed. To further investigate the molecular mechanism underlying the *Linc01559*-induced malignant phenotype, the subcellular localization of *Linc01559* was determined. It was found that *Linc01559* was predominantly localized to the nucleus (Figure 4a). An analysis of ribosome binding using the TranslatomeDB database (http://www.translatomedb.net (accessed on 15 August 2020)) revealed that *Linc01559* lacks protein-coding potential (Figure 4b). Additionally, the secondary structure of *Linc01559* was predicted using computational tools (https://www.tbi.univie.ac.at/RNA (accessed on 20 August 2020)) (Figure 4c). Next, candidate proteins that may interact with *Linc01559* were identified via RNA pulldown combined with mass spectrometry, followed by reverse verification using RNA immunoprecipitation (RIP). The target protein GRSF1 (Figure 4d–h), which interacts with *Linc01559*, was found to be positively associated with *Linc01559* expression (Figure 4i,j). GRSF1 is known to play a critical role in RNA transport, localization, stability, splicing, and post-transcriptional regulation, and is localized to both the nucleus and mitochondria. Previous studies have reported that GRSF1 can regulate lncRNA localization between the nucleus and mitochondria. An analysis of the relationships between GRSF1 and tumor stem cell markers revealed significant positive associations with *UBE2K*, *XRCC5*, *SNRPD3*, and *CDC123* (Appendix B Figure A2c), which was consistent with previous findings. In summary, *Linc01559* may influence the malignant phenotype of duodenal adenocarcinoma cells by regulating GRSF1 expression.

### 3.5. GRSF1 Is Highly Expressed in Acidic Environments and the Linc01559–GRSF1 Axis Regulates the Tumor Stem Cell Phenotype and Metastasis

**Figure 5:**
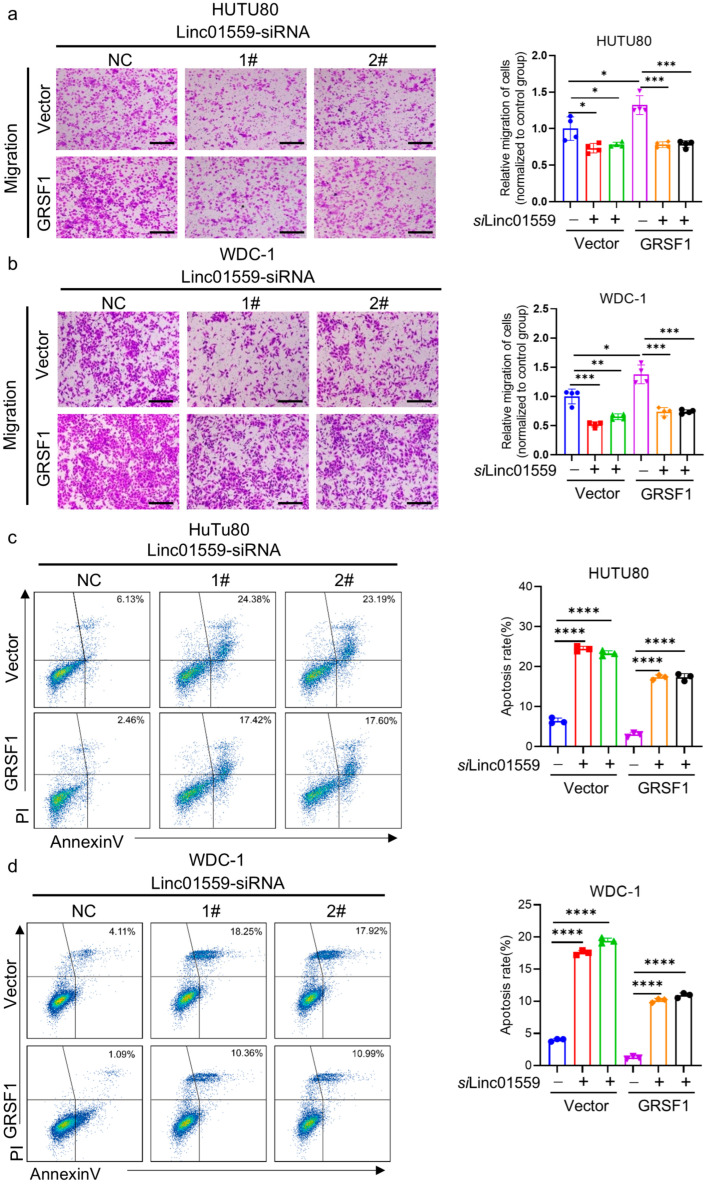
*Linc01559* affects the migration and apoptosis of duodenal adenocarcinoma through upregulating GRSF1. (**a**,**b**) Representative images (**left**) (scale bar, 100 µm) and quantified data (**right**) of the transwell assay showed the migration ability of control and *Linc01559*-knockdown duodenal adenocarcinoma cells treated with vector or GRSF1 plasmid; (**c**,**d**) representative images (**left**) and quantified data (**right**) of the flow cytometry showed the apoptosis of control and *Linc01559*-knockdown duodenal adenocarcinoma cells treated with vector or GRSF1 plasmid. Three independent experiments were performed to obtain the data in (**a**–**d**). The data are shown as the mean ±SD; *p*-values are shown * *p* < 0.05, ** *p* < 0.01, *** *p* < 0.001, and **** *p* < 0.0001.

**Figure 6:**
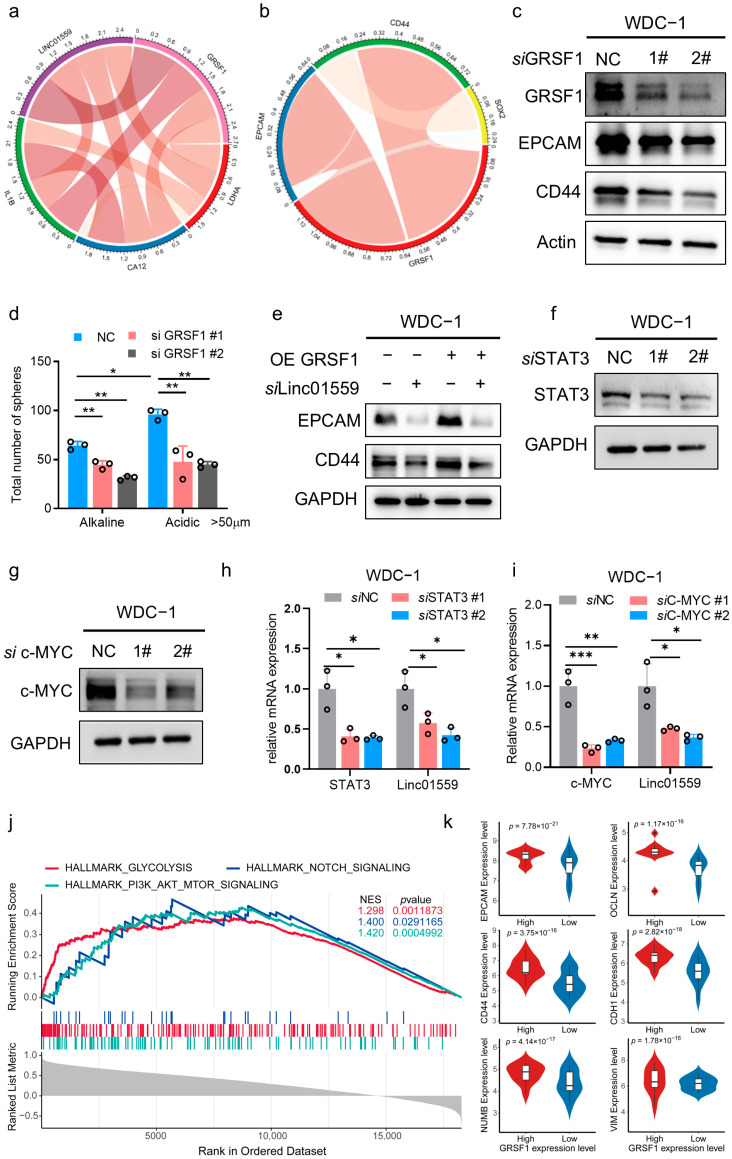
STAT3/c-MYC pathway accelerates the stemness of duodenal adenocarcinoma via the *Linc01559*–GRSF1 signal. (**a**) Correlation analysis among Linc01559, GRSF1, acidic environmental markers LDHA, CA12, and macrophage markers IL1B; (**b**) correlation analysis among GRSF1 and stemness marker EPCAM, CD44, and SOX2; (**c**) western blot of the protein expression of GRSF1, EPCAM, and CD44 in control and GRSF1-knockdown duodenal adenocarcinoma cells; (**d**) tumor spheres (with diameters larger than 50 µm) formed by control and GRSF1-knockdown duodenal adenocarcinoma cells cultured in alkaline and acidic conditions; (**e**) western blot of the protein expression of EPCAM and CD44 in control and Linc01559-knockdown duodenal adenocarcinoma cells treated with vector or GRSF1 plasmid; (**f**,**g**) western blot of the protein expression of STAT3 or c-MYC in control and STAT3-knockdown or c-MYC-knockdown duodenal adenocarcinoma cells; (**h**,**i**) qPCR of the RNA expression of *Linc01559* in control and STAT3-knockdown or c-MYC-knockdown duodenal adenocarcinoma cells; (**j**) GSEA of the cancer hallmark signals in GRSF1 high and GRSF1 low group from duodenal adenocarcinoma tumor tissues; (**k**) the mRNA expression of stemness markers EPCAM, CD44, NUMB, and EMT markers OCLN, CDH1, and VIM in GRSF1 high and GRSF1 low group. Three independent experiments were performed to obtain the data in (**d**,**h**,**i**). The data are shown as the mean ± SD; the Pearson correlation coefficient (r) and *p*-values are shown * *p* < 0.05, ** *p* < 0.01, and *** *p* < 0.001.

To investigate the role of the *Linc01559*–GRSF1 axis in regulating the malignant phenotype of duodenal adenocarcinoma cells, the expression profile of GRSF1 in these cells was first analyzed. GRSF1 was highly expressed in duodenal adenocarcinoma tissues compared with adjacent normal tissues (Appendix B Figure A2d). Migration assays confirmed that GRSF1 overexpression increased the migratory ability of duodenal cancer cells, whereas *Linc01559* knockdown significantly attenuated this effect (Figure 5a,b). Apoptosis assays revealed that GRSF1 overexpression inhibited the apoptosis of duodenal cancer cells, which aligned with the previous findings that showed increased duodenal cancer cell apoptosis following *Linc01559* knockdown (Figure 5c,d). Further analysis revealed significant positive correlations between *Linc01559*, GRSF1, and the acidic environment markers, LDHA, IL1B, and CA12 (Figure 6a and Appendix B Figure A1e). These findings indicate that GRSF1 is highly expressed in acidic environments and contributes to the regulation of the malignant phenotype of duodenal adenocarcinoma.

**Figure 7:**
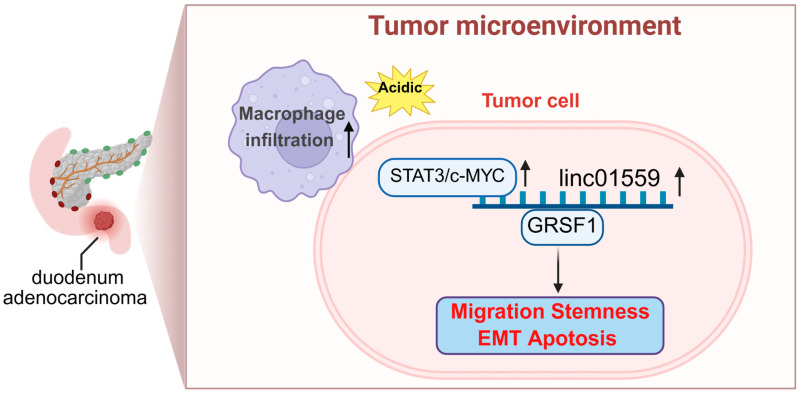
Schematic representation of the relationship of the STAT3/c-MYC–*Linc01559*–GRSF1 axis in an acidic tumor microenvironment with high macrophage infiltration. High macrophage infiltration and an acidic environment are two characteristics of the tumor microenvironment of duodenal cancer. STAT3 and c-MYC were upregulated in duodenal cancer and affected the expression of *Linc01559*, thereby promoting the malignant phenotype of duodenal cancer through the Linc01559–GRSF1 axis.

To further examine the role of the acidic environment–*Linc01559*–GRSF1 axis in the tumor stem cell phenotype, multiple correlation analyses were performed and revealed a significant positive association between GRSF1 and the tumor stem cell markers EPCAM and CD44 (Figure 6b). Knocking down GRSF1 expression reduced the expression of these markers and inhibited the ability of the acidic environment to enhance tumor stem cell self-renewal, as evidenced by decreased pellet formation (Figure 6c,d and Appendix B Figure A2f). Notably, GRSF1 overexpression partially reversed the inhibitory effect of Linc01559 knockdown on EPCAM and CD44 expression (Figure 6e). Further experiments revealed that knockdown of STAT3 and c-MYC expression inhibited *Linc01559* expression (Figure 6f–i). A GSEA revealed that GRSF1 participates in pathways associated with tumor malignancy (Figure 6j). In addition, high GRSF1 expression was associated with the upregulation of stem cell markers (including NUMB, CD44, and EPCAM) and EMT markers (including vimentin, cadherin-1, and occludin) (Figure 6k). These findings suggest that *Linc01559* interacts with GRSF1 to upregulate its expression, thereby promoting tumor cell migration, the stemness phenotype, and EMT as well as inhibiting apoptosis in duodenal adenocarcinoma (Figure 7).

## 4. Discussion

Duodenal adenocarcinoma exhibits marked metastatic propensity and aggressive local invasion, but therapeutic interventions for advanced-stage disease remain critically constrained, with current regimens showing limited clinical efficacy [4,32]. Therefore, a deeper understanding of the molecular mechanisms underlying the migration, invasion, and metastasis of duodenal adenocarcinoma cells is urgently needed. Schrock et al. identified *KRAS*, *TP53*, and *APC* mutations in duodenal adenocarcinoma, along with higher *CDKN2A/B* and *ERBB2/HER2* mutational frequencies compared to other small intestinal malignancies [33], whereas Yuan et al. revealed the critical role of the *WNT/β-catenin* pathway in duodenal adenocarcinoma progression through whole-exome sequencing [34]. Our transcriptomic analysis revealed significant upregulation of housekeeping genes (such as homeoboxB9) along with established gastrointestinal oncogenic markers [such as keratin (KRT)17, KRT7, mucin (MUC)5B, and KRT6B] in duodenal adenocarcinoma tissues. In the present study, it was demonstrated that lncRNAs are generally upregulated in duodenal adenocarcinoma. An enrichment analysis revealed that these lncRNAs are primarily involved in pathways associated with cell invasion, migration, apoptosis, and tumor stemness, suggesting a tumor-promoting role in disease progression.

The acidic TME drives immunosuppression, creating favorable conditions for tumor progression [35]. Mechanistically, an acidic environment promotes colorectal cancer metastasis by activating *PGC-1α*-dependent oxidative phosphorylation [22]. Moreover, an acidic environment upregulates insulin-like growth factor binding protein 6 expression, consequently facilitating immune evasion in glioblastoma [36]. The present study revealed that acidic environment markers were significantly upregulated in the tumor tissues of patients with duodenal adenocarcinoma, suggesting the presence of an acidic TME. TAMs are key regulators of the complex TME [37]. These TAMs, which are predominantly present in the acidic TME, promote tumor cell growth, angiogenesis, and metastasis. For instance, lactic acid from glioma cells stimulates TAM polarization to the M2 subtype via protein-coupled receptor 65 activation, which subsequently induces high mobility group box 1 secretion to increase glioma cell proliferation, migration, invasion, and EMT [38]. In the present study, a bioinformatics analysis of duodenal adenocarcinoma tissues revealed high expression of acidic environment markers and significant infiltration of macrophages, further highlighting the role of the acidic TME in tumor progression.

Recent studies have revealed that lncRNAs have a critical role in the polarization of tumor-associated macrophages (TAMs) and can serve as biomarkers for diagnosis and treatment. Specifically, the lncRNA PCAT6 regulates the microRNA (miR)-326/ Krüppel-like factor 1(KLF1) axis in non-small cell lung cancer (NSCLC) cells. The miR–326/*KLF1* axis inhibits TAM polarization and thereby influences metastasis and EMT [39], and *Linc01559* enhances vimentin expression, thereby increasing the complexity of vimentin–lncRNA interactions and promoting NSCLC metastasis [14]. The present study revealed that the knockdown of *Linc01559* markedly enhances the tumor malignant phenotypes of duodenal cancer cells, and the expression of *Linc01559* is positively correlated with macrophage infiltration and acidic environment, suggesting that *Linc01559* may act as a tumor promoter in the progression of duodenal adenocarcinoma and, thus, serve as a biomarker for clinical diagnosis.

GRSF1 is a member of the F/H family of heterogeneous ribonucleoproteins; it is widely expressed in eukaryotic cells; and localizes to the nucleus and cytoplasm, where it serves critical regulatory roles in RNA processing, transport, and translation in both the cytoplasm and mitochondria [40]. Previous studies have indicated that GRSF1 promotes malignant phenotypes such as cell proliferation, migration, and invasion of gastric [41], liver [20], and cervical cancer. However, its role in duodenal adenocarcinoma remains unclear. In the present study, it was determined that GRSF1 may function as a target protein of *Linc01559*. GRSF1 expression was significantly elevated in duodenal cancer tissues compared with normal tissues and was associated with tumor stemness, invasion, and apoptosis. The results suggested that GRSF1 may play a tumor-promoting role in duodenal adenocarcinoma under acidic conditions and is regulated by *Linc01559*. High expression of *Linc01559* and GRSF1 in duodenal cancer tissues indicates an increased risk of tumor recurrence and metastasis, suggesting that GRSF1 may represent a potential therapeutic target for duodenal cancer. However, further validation with larger sample sizes and additional studies are required.

## Data Availability

All mechanism diagrams are drawn through the BIORENDER website (https://app.biorender.com (accessed on 26 April 2025)). The original RNA-seq data have been uploaded to the Genome Sequence Archive (GSA; http://gsa.big.ac.cn/ (accessed on 9 May 2025)) and is accessible under the GSA numbers HRA011549. The total RNA extracted was from human tumor tissues that was obtained through ethical approval and patient consent.

## Abbreviations

DA	Duodenal adenocarcinoma
TME	tumor microenvironment
RNA-seq	RNA sequencing
LncRNA	long non-coding RNAs
GRSF1	G-rich sequence binding factor 1
STAT3	Signal transducer and activator of transcription 3
c-MYC	bHLH transcription factor
TAMs	tumor-associated macrophages
NSCLC	non-small cell lung cancer
EMT	epithelial-mesenchymal transition
PCA	principal component analysis
GSEA	Gene Set Enrichment Analysis
HIF1α	hypoxia inducible factor 1 subunit α
EPCAM	epithelial cell adhesion molecule
KRT	keratin
MUC	mucin
UBE2K	ubiquitin-conjugating enzyme E2 K
XRCC5	X-ray repair cross complementing 5
SNRPD3	small nuclear ribonucleoprotein D3 polypeptide
CDC123	cell division cycle 123

## Appendix A

**Table A1:** Primer sequences.

Name	Forward	Reverse
Primer Sequences for qRT-PCR		
ANO1	5′-CTGATGCCGAGTGCAAGTATG-3	5′-AGGGCCTCTTGTGATGGTACA-3′
KLK10	5′-CAAGGCGAACGGATGAGCA-3′	5′-GAGCACAGCGGTAGGGAAG-3′
KRT6B	5′-AGATTGCTCAGAGGAGCAGG-3′	5′-TGTAGGTTGGCACACTGCTT-3′
CDH3	5′-ATCATCGTGACCGACCAGAAT-3′	5′-GACTCCCTCTAAGACACTCCC-3′
HOXB	5′-AGGAGACGGAGGCTATTTTCA-3′	5′-GTCTGCTCGTTCCCATAAGGG-3
KRT17	5′-GGTGGGTGGTGAGATCAATGT -3′	5′-CGCGGTTCAGTTCCTCTGTC -3′
COL11A1	5′-ACCCTCGCATTGACCTTCC -3′	5′-TTTGTGCAAAATCCCGTTGTTT-3′
CXCL3	5′-CGCCCAAACCGAAGTCATAG-3′	5′-GCTCCCCTTGTTCAGTATCTTTT-3′
SLC2A1	5′-GGCCAAGAGTGTGCTAAAGAA-3′	5′-ACAGCGTTGATGCCAGACAG-3′
LAMC2	5′-GACAAACTGGTAATGGATTCCGC-3′	5′-TTCTCTGTGCCGGTAAAAGCC-3′
CEMIP	5′-CACGGTCTATTCCATCCACATC-3′	5′-GGTTCGCAAAACAATCGGCT-3′
DUOX2	5′-CTGGGTCCATCGGGCAATC-3′	5′-GTCGGCGTAATTGGCTGGTA-3′
MMP7	5′-GAGTGAGCTACAGTGGGAACA-3′	5′-CTATGACGCGGGAGTTTAACAT-3′
LCN2	5′-CCACCTCAGACCTGATCCCA-3′	5′-CCCCTGGAATTGGTTGTCCTG-3′
MUC5B	5′-GCCTACGAGGACTTCAACGTC-3′	5′-CCTTGATGACAACACGGGTGA-3′
GPRC5A	5′-ATGGCTACAACAGTCCCTGAT-3′	5′-CCACCGTTTCTAGGACGATGC-3′
CEACAM5	5′-CTGTCCAATGACAACAGGACC-3′	5′-ACGGTAATAGGTGTATGAGGGG-3′
SLC6A14	5′-ACCGTGGTAACTGGTCCAAAA-3′	5′-CGCCTCCACCATTGCTGTAG-3′
INHBA	5′-CCTCCCAAAGGATGTACCCAA-3′	5′-CTCTATCTCCACATACCCGTTCT-3′
TACSTD2	5′-ACAACGATGGCCTCTACGAC-3′	5′-GTCCAGGTCTGAGTGGTTGAA-3′
CLDN	5′-AGCTGCAAAATGTACGACTCG-3′	5′-GGAGACCACCATTAGGGCTC-3′
LNC CCAT1	5′-CCATAATGTAGAATCAGTGGAAGC-3′	5′-TCTCATAGCAGCACAAACCCT-3′
LNC RP11-357H14.17	5′-CCCACTCCCTTTCTTCCTTGA-3′	5′-CGCCTGTAATGAACCCTGTGA-3′
LNC LUCAT1	5′-TACCTGTCCTGCGTGTTGAA-3′	5′-TCTTTGGGTAATTTTTGGGATCT-3′
LNC HOTTIP	5′-GTATCGGGCAAAGGTGGAAAA-3′	5′-ATGAAAAGGGAGCAAGGTCGT-3′
LNC RP3-416H24.1	5′-TTGCCTTCAATGAGATGACCTTC-3′	5′-GCTTTCCCCTCTGGAGACTAA-3′
LNC 01559	5′-TCTCCTTTTCTCACTCCTCCC-3′	5′-TTCCTCCTCTGGTTTCTCATG-3′
LNC CTD-2377D24.6	5′-GAGGCAGCAGTCAATACCCAC-3′	5′-ATCTCAATGGAAGAATGCGACA-3′
LNC FEZF1	5′-TTCAGTCAAGAAGGCAGGTAA-3′	5′-GATGTCTAACAGAAAGGCAGTG-3′
LINC01021	5′-CGAGACCATCTTGGCTAACACT-3′	5′-TCGGCTCACTACAAGCTCTGC-3′
LNC HOXA-AS3	5′-CTGGAAAGGTCGGTTGTAAAG-3′	5′-ATAGCGACTTTTGGGATAGTTTGC-3′
LNC HOXB-AS3	5′-TTACTGGACTTGGAGGGAGGG-3′	5′-ATAGCGACTTTTGGGATAGTTTGC-3′
STAT3	5′-CAGCAGCTTGACACACGGTA-3′	5′-AAACACCAAAGTGGCATGTGA-3′
c-MYC	5′-GGCTCCTGGCAAAAGGTCA-3′	5′-CTGCGTAGTTGTGCTGATGT-3′

**Table A2:** Clinical statistics of samples from RNA-seq.

No	Gebder	Age	Location	Differentiation Grade	Therapy	CA199 u/mL	CEA ug/L
1	Female	66	Ampullae	Moderately differentiated	None	157.2	2.1
2	Male	69	descending part	Moderately differentiated	None	157.5	5.5
3	Male	52	Superior par	Moderately differentiated	None	12.27	2.41
4	Female	62	Papilla	Moderately differentiated	None	313.9	1.58
5	Male	62	horizontal part	Moderately differentiated	None	10.43	1.3
6	Male	70	Ampullae	Moderately differentiated	None	17.71	9.59
7	Male	55	descending part	Poorly differentiated	None	0.69	2.85
8	Male	46	Ampullae	Moderately differentiated	None	21.56	3.19
9	Male	57	Ampullae	Moderately differentiated	None	24.15	3.14
10	Male	66	Ampullae	Moderately differentiated	None	58.6	6.8
11	Female	46	Papilla	Moderately differentiated	None	112.5	4.73

**Table A3:** Antibody.

Antibody	Source	Identifier
Anti-GRSF1 antibody	Signalway	54507
Anti-EpCAM antibody [E144]	abcam	ab32392
Anti-Actin antibody	Proteintech	HRP-6609
Anti-STAT3 antibody	SCBT	SC-8019
Anti-GAPDH antibody	Proteintech	60004-1-Ig
Anti-CD44 antibody [EPR1013Y]	abcam	ab51037
Anti-c-MYC antibody	proteintech	10828-1-AP
HRP-conjugated Goat Anti-Mouse IgG(H + L) HRP-conjugated Goat Anti-Rabbit IgG(H + L)	Proteintech Proteintech	SA00001-1 SA00001-2

**Table A4:** Reagent.

Reagents	Source	Identifier
EGF	Peprotech	AF-100-15
FGF-basic	Peprotech	100-18B
HEPES	Gibco	15630080
PIPES	Sigma	P1851
Falcon^®^ 40 µm Cell Strainer	Corning	352340
B-27™ Supplement (50×), minus vitamin A	Gibco	12587010
Collagenase, Type I, powder	Gibco	17100017
StemPro™ Accutase™ Cell Dissociation	Gibco	A1110501
ViaFect™ Transfection Reagent	promega	E4981

## Appendix B

Figure A1 and Figure A2.
