# Correction: Huang et al. Duodenal Adenocarcinoma Is Characterized by Acidity, High Infiltration of Macrophage, and Activated Linc01559–GRSF1 Axis. *Biomedicines* 2025, *13*, 1611

**DOI:** 10.3390/biomedicines13102531

**Published:** 2025-10-17

**Authors:** Xinxin Huang, Ying Shi, Zekun Liu, Yihang Wu, Xiaotong Luo, Dongwen Chen, Zhengyu Wei, Chong Chen, Huaiqiang Ju, Xiaojian Wu, Xuanhui Liu, Zhanhong Chen, Peishan Hu

**Affiliations:** 1 Department of General Surgery (Colorectal Surgery), The Sixth Affiliated Hospital of Sun Yat-sen University, Sun Yat-sen University, Guangzhou 510630, China; huangxx97@mail2.sysu.edu.cn (X.H.);; 2 Guangdong Provincial Key Laboratory of Colorectal and Pelvic Floor Diseases, Guangdong Institute of Gastroenterology, The Sixth Affiliated Hospital of Sun Yat-sen University, Sun Yat-sen University, Guangzhou 510630, China; 3 Department of Radiology, Sun Yat-sen University Cancer Center, Sun Yat-sen University, Guangzhou 510220, China; 4 Sun Yat-sen University Cancer Center, State Key Laboratory of Oncology in South China, Guangdong Provincial Clinical Research Center for Cancer, Sun Yat-sen University, Guangzhou 510220, China; 5 Department of Medical Oncology and Guangdong Key Laboratory of Liver Disease, The Third Affiliated Hospital of Sun Yat-sen University, Sun Yat-sen University, Guangzhou 510630, China

## Correction

In the original publication [1], a retracted reference “19. Yang, Z.; Sun, Q.; Guo, J.; Wang, S.; Song, G.; Liu, W.; Liu, M.; Tang, H. GRSF1-mediated MIR-G-1 promotes malignant behavior and nuclear autophagy by directly upregulating *TMED5* and *LMNB1* in cervical cancer cells. *Autophagy* **2019**, *15*, 668–685. https://doi.org/10.1080/15548627.2018.1539590.” was cited. The citation has now been removed in the Section 1 Introduction, Paragraph 3, and in the Section 4 Discussion, Paragraph 4, which now read as follows:

G-rich sequence binding factor 1 (GRSF1) is an RNA-binding protein involved in transcriptional regulation [18]. Studies have indicated that dysregulated GRSF1 contributes to tumor progression [19]. Notably, GRSF1 enhances the stability of *YY1* mRNA, thereby promoting hepatocarcinogenesis [20]. However, the upstream regulatory mechanisms governing GRSF1 in duodenal cancer remain unclear.

GRSF1 is a member of the F/H family of heterogeneous ribonucleoproteins; it is widely expressed in eukaryotic cells; and localizes to the nucleus and cytoplasm, where it serves critical regulatory roles in RNA processing, transport, and translation in both the cytoplasm and mitochondria [40]. Previous studies have indicated that GRSF1 promotes malignant phenotypes such as cell proliferation, migration, and invasion of gastric [41], liver [20], and cervical cancer. However, its role in duodenal adenocarcinoma remains unclear. In the present study, it was determined that GRSF1 may function as a target protein of *Linc01559*. GRSF1 expression was significantly elevated in duodenal cancer tissues compared with normal tissues and was associated with tumor stemness, invasion, and apoptosis. The results suggested that GRSF1 may play a tumor-promoting role in duodenal adenocarcinoma under acidic conditions and is regulated by *Linc01559*. High expression of *Linc01559* and GRSF1 in duodenal cancer tissues indicates an increased risk of tumor recurrence and metastasis, suggesting that GRSF1 may represent a potential therapeutic target for duodenal cancer. However, further validation with larger sample sizes and additional studies are required.

With this correction, the order of some references has been adjusted accordingly. The authors state that the scientific conclusions are unaffected. This correction was approved by the Academic Editor. The original publication has also been updated.
